# Hydrolyzed collagen-modified bacterial cellulose loaded with tea tree oil for antibacterial activity against acne-associated bacteria

**DOI:** 10.1039/d5ra09816e

**Published:** 2026-02-17

**Authors:** Taiwo Salawudeen, Juntratip Jomrit, Sirikanya Kaewpradit, Nuttikarn Nokkaew, Chutima Jantarat

**Affiliations:** a School of Pharmacy, Walailak University Thasala Nakhon Si Thammarat 80160 Thailand chutima.ja@wu.ac.th; b College of Graduate Studies, Walailak University Thasala Nakhon Si Thammarat 80160 Thailand; c Drug and Cosmetics Excellence Center, Walailak University Thasala Nakhon Si Thammarat 80160 Thailand

## Abstract

Acne is a chronic inflammatory skin disorder commonly treated with topical antibiotics, whose long-term use is limited by antimicrobial resistance and adverse effects. Tea tree oil (TTO) is a natural antimicrobial agent effective against acne-associated bacteria; however, its clinical application is hindered by volatility, oxidative instability, and skin irritation at high concentrations. In this study, a novel biopolymer-based delivery system was developed using bacterial cellulose modified with hydrolyzed collagen (BC/HC) to enhance the loading, stability, and antibacterial efficacy of TTO for potential acne therapy. BC/HC composites were prepared *via* both *in situ* and *ex situ* collagen modification approaches and comprehensively characterized for their physicochemical properties. *In situ* modification significantly reduced crystallinity (from ∼81% in native BC to ∼76% at the highest HC content), increased porosity, and improved water-holding capacity, resulting in markedly enhanced TTO loading efficiency-up to eightfold compared with unmodified BC when loaded in the swollen state. The TTO-loaded BC/HC composites exhibited a desirable biphasic release profile with an initial burst followed by sustained release. Concentration-dependent antibacterial activity against *Staphylococcus aureus* and *Cutibacterium acnes* was demonstrated through disc diffusion and time-kill kinetic assays, which showed no detectable colonies within 2–3 h at the highest TTO loadings. Stability studies showed that high terpinen-4-ol content (>90%) and approximately 70% antibacterial activity were retained after three months under both normal and accelerated storage conditions for composites with high TTO loading, significantly outperforming conventional substrates. Overall, *in situ* HC-modified BC represents a promising, natural, and sustainable delivery platform for TTO, offering enhanced loading capacity, controlled release, potent antibacterial activity, and improved stability, with strong potential as a topical antimicrobial platform active against acne-associated bacteria.

## Abstract 2

Preparation and antibacterial evaluation of TTO-loaded BC/HC composites.

## Graphical abstract

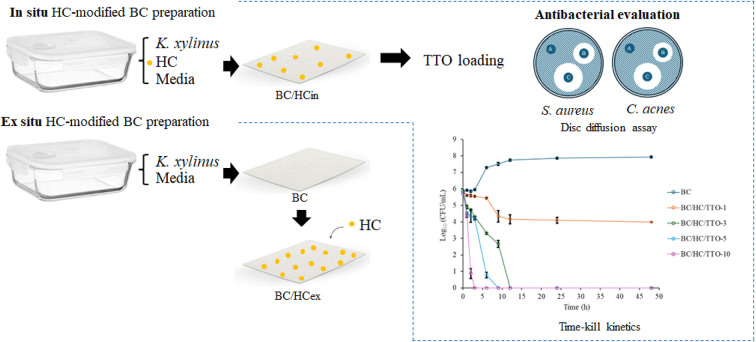

## Introduction

Acne is one of the most prominent dermatological disorders and is characterized as a chronic inflammatory condition of the pilosebaceous unit that affects millions of individuals worldwide.^1^ The global prevalence of acne is approximately 9.4% across all age groups,^2^ with the highest burden occurring during adolescence, where nearly 85% of individuals experience the condition.^3^ Acne has far-reaching impacts beyond its physical manifestations, often diminishing quality of life and contributing to emotional distress, pain, discomfort, permanent scarring, reduced self-esteem, and, in severe cases, anxiety and depression.^4–6^ The pathogenesis of acne is multifactorial, involving increased sebum production, abnormal follicular keratinization, and microbial colonization, particularly by *Staphylococcus aureus* and *Cutibacterium acnes* (formerly *Propionibacterium acnes*), which drive inflammation and lesion development.^7,8^

Topical therapies remain the cornerstone of acne management due to their minimal systemic absorption, reduced systemic side effects, and enhanced delivery of active agents to the pilosebaceous unit. Commonly prescribed topical treatments include benzoyl peroxide, topical retinoids, and topical antibiotics such as clindamycin or erythromycin, which are frequently used in combination regimens. However, the long-term effectiveness of these therapies is increasingly challenged by the rise of antimicrobial resistance, largely driven by excessive antibiotic use.^9^ These treatments may also cause undesirable side effects, including dryness, irritation, peeling, burning sensations, and dyschromia.^10,11^ Consequently, there is growing interest in sustainable alternative treatments with improved safety and reduced resistance potential.

Plant-derived essential oils have emerged as promising alternatives due to their natural antimicrobial, antioxidant, and anti-inflammatory properties.^12^ Among them, tea tree oil (TTO), extracted from the leaves of *Melaleuca alternifolia*, has shown significant antibacterial activity against acne-associated bacteria and was clinical efficacy in reducing acne lesion count and severity.^13–16^ Its antibacterial effect, attributed mainly to terpinen-4-ol and supported by minor constituents including terpinolene, α-terpinene, and α-terpineol, is active against both *S. aureus* and *C. acnes*.^17,18^ Reported minimum inhibitory concentration (MIC) and minimum bactericidal concentration (MBC) values for TTO range from 0.2–1% (v/v) for *S. aureus* and 0.25–0.5% (v/v) for *C. acnes*.^19–22^ Despite these benefits, TTO can cause skin irritation at high concentrations^23,24^ and is prone to oxidative degradation triggered by light, oxygen, humidity, and heat, leading to reduced antibacterial activity and increased irritancy.^25–28^ Stabilizing and controlling the release of TTO is therefore essential for optimizing its therapeutic potential.

Biopolymers have gained significant attention as versatile materials in biomedical applications.^29^ Bacterial cellulose (BC), a highly pure β-1,4-glucan produced by *Komagataeibacter xylinus* and related species, has emerged as a promising material for drug delivery due to its purity, mechanical strength, biocompatibility, nanofibrous porosity, renewability, and low production cost.^30,31^ Its hydrophilic nature, however, limits the efficient incorporation of hydrophobic compounds such as TTO. Previous studies have attempted to address this limitation by using emulsions or ethanol-based solutions for loading essential oils into BC,^32–34^ but these approaches may introduce surfactant-related irritation, confound antibacterial evaluation, or require impractically high oil concentrations.

Modification of BC with collagen has emerged as a promising strategy to enhance loading capacity for hydrophobic drugs. Collagen-altered BC exhibits modified pore structure and fibrous architecture, improving the incorporation of pharmaceuticals and proteins.^35–37^ Additionally, collagen itself contributes therapeutic benefits for the skin, including promoting skin repair, hydration, elasticity, and reducing inflammation, making it a suitable adjunct for acne treatment.^38,39^ Although BC-based systems have previously been explored as carriers for antimicrobial essential oils, primarily in wound-dressing applications, the influence of different collagen modification strategies (*in situ versus ex situ*) on essential oil loading efficiency and stability has not been systematically investigated. Moreover, there is limited evidence regarding the stabilization of TTO within natural biopolymer matrices and the preservation of its antibacterial efficacy against acne-associated bacteria.

Therefore, this study aimed to develop TTO-incorporated BC modified with hydrolyzed collagen (BC/HC) for potential topical application in acne management. BC/HC composites were prepared using both *in situ* and *ex situ* approaches and systematically characterized in terms of their morphological, physical, and chemical properties, as well as water absorption capacity and crystallinity. Based on these evaluations, the most suitable composite was selected for further formulation studies. The TTO loading capacity of the selected BC/HC composite was subsequently quantified, and the resulting TTO-loaded materials were evaluated for antimicrobial activity against *S. aureus* and *C. acnes*. In addition, system stability, including TTO retention and the preservation of antibacterial activity, was assessed. This work provides insight into the development of a natural, biopolymer-based delivery system with enhanced TTO loading efficiency and stability, offering sustained antimicrobial activity against acne-associated bacteria under the tested conditions.

## Materials and methods

### Chemicals

Tea tree oil (TTO; extra grade, terpinen-4-ol ≥ 43%) was obtained from Chanjao Longevity Co., Ltd. (Thailand). The terpinen-4-ol primary reference standard (≥95%) was purchased from HWI Pharma Services GmbH (Germany). Components of the Hestrin-Schramm (HS) media, including glucose, bacto peptone, yeast extract, disodium hydrogen phosphate, and citric acid, were sourced from Sigma-Aldrich Co., Ltd. (USA). Mueller-Hinton broth (MHB), Mueller-Hinton agar (MHA), brain-heart infusion (BHI) agar, BHI broth, and cation-adjusted Mueller-Hinton broth (CAMHB) were obtained from HiMedia Laboratories Pvt., Ltd. (India). Hydrolyzed collagen (HC) was purchased from PC Drug Center Co., Ltd. (Thailand). All other chemicals and reagents were of analytical grade and used without further purification.

### Microorganisms

*Komagataeibacter xylinus* (TISTR 107), used for BC production, was obtained from the Thailand Institute of Scientific and Technological Research (TISTR), Pathum Thani, Thailand. *Cutibacterium acnes* (DMST 14916) and *Staphylococcus aureus* (ATCC 25923), employed for antibacterial activity evaluation, were sourced from the Department of Medical Sciences, Ministry of Public Health (Thailand), and the American Type Culture Collection (USA), respectively. The protocols related to the use of microorganisms were approved by the Institutional Biosafety Committee, Walailak University (WU-IBC-66-069).

### Biosynthesis of BC

BC was produced by cultivating *K. xylinus* in HS media composed of 2% (w/v) glucose, 0.5% (w/v) bacto peptone, 0.5% (w/v) yeast extract, 0.115% (w/v) citric acid, and 0.27% (w/v) disodium phosphate. Bacterial activation was initiated by inoculating the lyophilized culture into a 10-mL tube containing HS media and incubating it statically at 30 °C for 7 days. For seed culture preparation, 10 mL of activated culture was transferred into 250-mL glass bottles containing 100 mL of HS media and incubated under static conditions at 30 °C for an additional 7 days. BC production was carried out by inoculating 25 mL of seed culture into a 500-mL glass container containing 225 mL of HS media (10% v/v inoculum) and incubating statically at 30 °C for 9 days. A BC pellicle formed at the air–liquid interface and was harvested for purification. The pellicles were rinsed with distilled water to remove residual media, treated with 0.1 M sodium hydroxide until discoloration ceased, and subsequently heated at 80 °C for 45 min to ensure removal of remaining cells. The samples were washed repeatedly with distilled water until neutral pH was achieved. Purified BC was stored at 4 °C until further use.

### Preparation of HC-modified BC (BC/HC composites)

#### *In situ* modification

Different amounts of HC (0.1, 0.5, and 1 g) were added together with 25 mL of *K. xylinus* seed culture into a 500-mL glass container containing 225 mL of HS media. The mixtures were statically incubated at 30 °C for 9 days to yield BC/HC composites (BC/HCin), designated as BC/HCin-0.1, BC/HCin-0.5, and BC/HCin-1, corresponding to the HC amount in the culture media. The resulting composites were washed and treated following the same procedure described for BC purification. The composites were stored in sealed glass containers at 4 °C until further use. For characterization, the composites were dried at 45 °C for 7 h to obtain dry films, which were subsequently stored in a desiccator.

#### *Ex situ* modification

Purified BC pellicles obtained from “Biosynthesis of BC” were compressed between two filter papers using a spatula to remove approximately half of the water content. The pellicles were then immersed in 225 mL of aqueous HC solution (0.1, 0.5, or 1 g) and incubated at room temperature (25 ± 1 °C) under gentle stirring for 24 h. The resulting BC/HC composites (BC/HCex) were designated as BC/HCex-0.1, BC/HCex-0.5, and BC/HCex-1, according to the HC amount in the immersion solution. Excess HC was removed by gently blotting the surface with tissue paper. The composites were stored in sealed glass containers at 4 °C until further use. For characterization, the composites were dried at 45 °C for 7 h to obtain dry films, which were subsequently stored in a desiccator.

Incubation temperature, medium composition, incubation duration, and static or stirred conditions were strictly controlled and kept constant across all batches and composite types to ensure reproducibility.

### Characterization of BC/HC composites

#### Fourier transform infrared (FTIR) spectroscopy

FTIR spectra of BC/HC composites, including TTO-loaded BC/HC composites, were recorded using a Bruker Tensor 27 FTIR spectrometer (Bruker Optics, Ettlingen, Germany) in attenuated total reflectance (ATR) mode. Spectra were acquired over the range of 4000–500 cm^−1^ with a resolution of 4 cm^−1^, using 32 scans per sample. Baseline correction was applied to all spectra.

#### Scanning electron microscopy (SEM)

The surface and cross-sectional morphology of BC/HC composites were examined using SEM and compared with pure BC. For cross-sectional analysis, samples were immersed in liquid nitrogen and fractured to obtain a neat surface. All samples were mounted on carbon tape, sputter-coated with a thin layer of gold to enhance conductivity and examined under high vacuum at an accelerating voltage of 5–10 kV. Working distances ranged from 3.2 to 8.0 mm, with magnifications of 5000× for cross-sections and 200 00× for surface morphology.

#### Differential scanning calorimetry (DSC)

Thermal characteristics of BC/HC composites, including TTO-loaded BC/HC composites, was evaluated using a differential scanning calorimeter (DSC 6000; PerkinElmer, Waltham, MA, USA). Approximately 5.0–5.5 mg of each sample was placed in an aluminum pan and sealed. An empty pan was used as reference. Samples were scanned from 0 °C to 445 °C at a heating rate of 10 °C min^−1^ under a nitrogen flow of 20 mL min^−1^.

#### X-ray diffraction (XRD)

XRD analysis was performed to determine the crystallinity of BC/HC composites obtained *via* the *in situ* method, in comparison with pure BC. Samples were analyzed using an Empyrean X-ray diffractometer (PANalytical, Netherlands) with Ni-filtered CuKα radiation (*λ* = 0.154 nm) at 40 kV and 30 mA. Data were collected over 2*θ* = 5–90° with a step size of 0.026° and a step time of 70.125 s; samples were rotated with a period of 1 s. Crystallinity (%) was calculated using:1
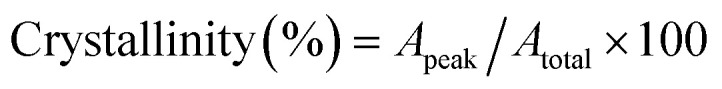
where *A*_peak_ is the area under crystalline peaks, and *A*_total_ is the total peak area. All samples were stored in a desiccator prior to analysis.

#### Mechanical testing

Tensile strength of BC/HC composites (both *in situ* and *ex situ*) and pure BC was evaluated according to ASTM D882.^40^ Samples were cut into rectangles (1 × 2 cm) and tested using a universal testing machine (Lloy, UK) at a crosshead speed of 1 mm min^−1^ until fracture. Maximum force (*F*_max_) was recorded, and tensile strength (*σ*_T_) was calculated as:

2 *σ*_*T*_ = *F*_max_/*A*

where *A* is the cross-sectional area. Three replicates were tested for each sample type.

#### Water holding capacity (WHC)

WHC was assessed by cutting samples into 2 × 2 cm pieces, weighing them (*W*_dry_), and immersing in 30 mL of distilled water at 25 ± 1 °C. After defined intervals (1–24 h), excess water was removed using filter paper, and samples were weighed again (*W*_wet_). WHC was calculated as:3
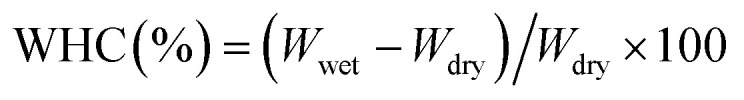


All measurements were performed in triplicate.

#### Water retention value (WRV)

WRV, an empirical measure of water retention, was determined using swollen BC and BC/HC composites cut into 2 × 2 cm samples. Samples were dried in a hot air oven at 45 °C. The WRV was calculated using:4
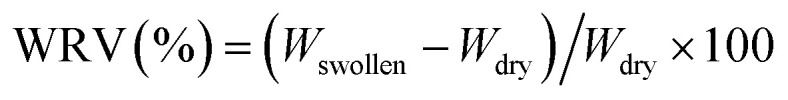
where *W*_swollen_ and *W*_dry_ represent the weights of the swollen and dry samples, respectively. All measurements were performed in triplicate.

### Preparation of TTO-loaded BC/HC composites

The loading efficiency of TTO into BC/HC composites prepared *via* both *in situ* and *ex situ* modification methods was initially compared. A 10% (v/v) TTO loading solution was prepared by mixing 5 mL of TTO with 45 mL of ethanol, followed by stirring for 2 h at room temperature (25 ± 1 °C). To ensure comparability among different BC/HC composites, all samples were standardized prior to TTO loading. Dried BC/HC composite films were cut into identical dimensions (4 × 4 cm), and their initial dry weights were recorded for subsequent normalization of TTO loading capacity. Each membrane was then fully immersed in the TTO solution under agitation at 250 rpm for 24 h. After loading, the samples were dried in a hot-air oven at 45 °C for 6 h to obtain the dry TTO-loaded BC/HC composites. Throughout the loading procedure, agitation speed, immersion time, solution composition, and temperature were kept constant to ensure consistent and reproducible uptake of TTO within the BC/HC matrix.

TTO loading efficiency was also evaluated using BC/HC composites in a swollen form, examined using *in situ*-prepared BC/HC composites. The swollen composites were partially compressed to remove free water, reducing their weight by approximately 50%, in order to facilitate enhanced TTO uptake upon re-swelling. This compression procedure was applied uniformly to all samples to minimize variations in hydration level and thickness prior to loading. The compressed composites were then immersed in the TTO solution, and the loading process was conducted in the same manner as for the dried composites as described above. During agitation at 250 rpm for 24 h, the compressed composites gradually re-swelled to their original dimensions. Pure BC films were similarly loaded with TTO to use as controls. All TTO-loaded samples were stored in sealed amber bottles until further use.

Based on the TTO loading capacity results, *in situ*-modified BC/HC composites exhibited superior loading efficiency compared to *ex situ*-modified samples. Among these, BC/HCin-1, containing the highest HC content evaluated at a fixed TTO concentration, showed the highest TTO loading capacity. Therefore, BC/HCin-1 was selected for subsequent evaluations, including *in vitro* release studies, antibacterial testing, time-kill kinetics, and stability assessments. This composite, used in its swollen form, was loaded with TTO by immersion in ethanol-based solutions containing 1%, 3%, 5%, and 10% (v/v) TTO. The resulting samples were designated as BC/HC/TTO-1, BC/HC/TTO-3, BC/HC/TTO-5 and BC/HC/TTO-10, corresponding to their respective TTO loading concentrations. A vehicle control, designated as BC/HC/EtOH, was prepared by immersing the composite in ethanol without TTO using the same procedure and was included in antibacterial activity evaluations.

### Quantification of TTO content in TTO-loaded BC/HC composites

TTO content in TTO-loaded BC/HC composites and TTO-loaded BC was quantified using gas chromatography-mass spectrometry (GC-MS, Agilent 7890b coupled with Q-TOF 7200, Agilent Technologies, Santa Clara, CA, USA) equipped with an HP-5MS Ultra Inert column (30 m × 0.25 mm × 0.25 µm). A 1 × 1 cm sample was extracted with 2 mL of hexane using sonication for 15 min at 25 ± 1 °C. The extract was diluted with hexane, and 1 µL was injected (split ratio 1 : 100). The oven program was: 50 °C for 10 min, ramped to 250 °C at 4 °C min^−1^. Helium was used as the carrier gas at 1.0 mL min^−1^. Terpinen-4-ol was used as a marker compound. The loading capacity of terpinen-4-ol was calculated relative to the dry mass of the composite using the following equation:5
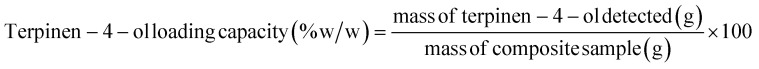


All measurements were performed in triplicate.

### *In vitro* release study of TTO-loaded BC/HC composites

TTO release was studied using a Franz diffusion cell (FDC-6, Logan Instruments Corp., USA) with a receptor volume of 12 mL and a diffusion area of 0.78 cm^2^. A cellulose acetate membrane (0.45 µm pore size) separated the donor and receptor compartments, with 50% ethanol in water as the receptor medium. Solubility of TTO in 50% ethanol was confirmed to be 9.32 ± 3.20 mg mL^−1^, which was sufficient to maintain sink conditions throughout the experiment and ensure diffusion under steady-state conditions. Membrane samples of identical dimensions (∼2 × 2 cm) were used for all experiments. Each sample was placed over the receptor compartment, covered with the donor compartment, and sealed with parafilm. The receptor medium was stirred at 600 rpm at 32 ± 1 °C. Samples (0.5 mL) were withdrawn at intervals from 0.5–24 h and replaced with fresh medium. TTO content was analyzed by gas chromatography-flame ionization detector (GC-FID) with HP-5MS column, 30 m × 0.25 mm × 0.25 µm and helium as carrier gas at 2 mL min^−1^. Injector: split mode (5 : 1), 1 µL injection. Oven program: 50 °C for 3 min, ramp to 200 °C at 10 °C min^−1^, held 7 min. FID temperature: 280 °C, hydrogen 40 mL min^−1^, air 400 mL min^−1^. Release data were expressed as cumulative terpinen-4-ol release *versus* time. All experiments were performed in triplicate.

### Determination of antibacterial activity

The antibacterial activity of TTO-loaded BC/HC composites against *S. aureus* and *C. acnes* was evaluated using the disc diffusion assay. Composite films were punched into discs of uniform diameter (6 mm) to ensure material comparability. All samples were UV-sterilized for 30 min. Clindamycin (CLI, 2 mg/disc) was used as positive controls as a clinically relevant reference antibiotic, while BC and BC/HC/EtOH served as negative and vehicle controls, respectively. Bacterial suspensions were prepared at a standardized concentration of approximately 1 × 10^8^ CFU mL^−1^ and evenly spread on MHB plates for *S. aureus* and BHI agar plates for *C. acnes*. Samples were placed on the inoculated surfaces and incubated at 37 °C for 24 h under aerobic conditions (*S. aureus*) or 48 h under anaerobic conditions (*C. acnes*).

The antibacterial activity was quantified by measuring the diameter of the zone of inhibition (ZOI) in millimetres. All experiments were conducted in triplicate, and results are reported as mean ± standard deviation. As disc dimensions and experimental conditions were standardized, differences in ZOI were attributed to variations in TTO content and release behaviour.

### Time-kill kinetics of TTO-loaded BC/HC composites

Time-kill kinetics were evaluated using the broth macro-dilution method. Bacterial inocula were prepared at an initial concentration of 5 × 10^5^ CFU mL^−1^ in CAMHB for *S*. *aureus* and in BHI broth for *C*. *acnes*. Composite films were punched into discs of uniform diameter (6 mm) and individually incubated with 400 µL of the bacterial suspension in sterile tubes at 37 °C under static conditions. Incubation was performed under aerobic incubations for *S. aureus* and anaerobic conditions for *C. acnes*. At predetermined time points 0–24 h for *S. aureus* and 0–48 h for *C. acnes*), 100 µL aliquots were withdrawn, serially diluted ten-fold in the corresponding broth medium, and plated onto MHA or BHI agar. Plates were incubated under the same atmospheric conditions as described above, after which colonies were enumerated. Viable bacterial counts were calculated as CFU mL^−1^ based on the plated volume and dilution factor. Bacterial growth in the control groups was assumed to follow standard exponential kinetics. CFU counts were determined at predefined time points and expressed as CFU mL^−1^. All experiments were performed in triplicate.

### Stability studies of TTO-loaded BC/HC composites

BC/HC/TTO-1 and BC/HC/TTO-10 composites were selected for stability evaluation. Samples (4 × 4 cm) were stored in sealed amber bottles under the test conditions: 30 ± 2 °C/75 ± 5% RH and 40 ± 2 °C/75 ± 5% RH, according to ASEAN stability guidelines.^41^ Samples were analyzed after 1 and 3 months to determine TTO content and antibacterial activity, and results were compared with freshly prepared samples (day 0). TTO content was quantified by extracting 1 × 1 cm pieces of each composite in 5 mL ethanol using 20 min sonication, followed by GC-FID analysis as described in Section “*In vitro* release study of TTO-loaded BC/HC composites”. Retained antibacterial activity against indicator pathogens was determined using the disc diffusion assay (Section “Determination of antibacterial activity”). Retained microbial activity was calculated as percentage relative to the initial ZOI (day 0), ensuring consistent comparison across storage conditions and time points using the following equation:

6 Retained antibacterial activity (%) = ZOI at time *t*/ZOI at day0 × 100

All experiments were performed in triplicate.

## Results and discussion

### Preparation of BC and BC/HC composites

BC was successfully produced *via* static fermentation using *K*. *xylinus* (TISTR 107). After nine days of incubation, a thick pellicle formed at the air–liquid interface, confirming BC biosynthesis. This observation aligns with previous reports indicating that *K. xylinus* readily forms surface pellicles under static culture conditions.^42^ The yield of fully swollen BC was 34.80 ± 0.93 g (10 × 15 cm), corresponding to 139.20 ± 3.72 g L^−1^, which is consistent with the findings of Ammar *et al.* (2022),^43^ who reported a yield of 139.4 g L^−1^.

**Fig. 1:**
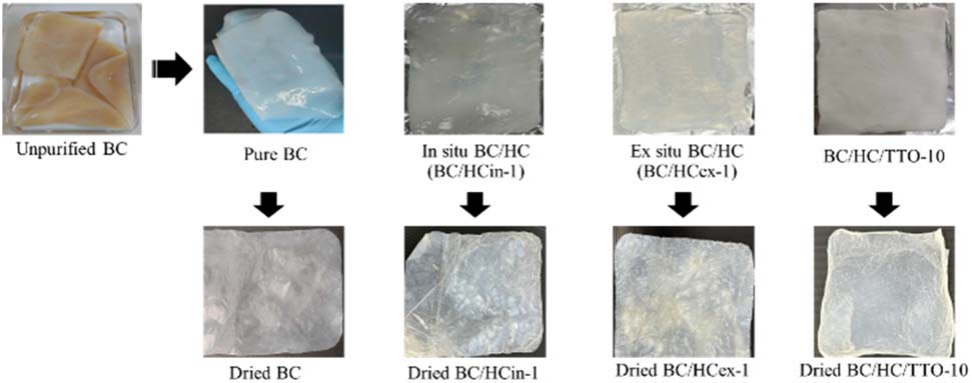
Physical appearance of BC before and after purification, BC modified with HC by *in situ* and *ex situ* methods (BC/HCin-1 and BC/HCex-1 shown as representatives), and BC/HC composite loaded with TTO (BC/HC/TTO-10 shown as representatives).

BC modification with HC was successfully achieved using both *in situ* and *ex situ* approaches. In the *in situ* method, HC was added directly to the culture media, producing a softer and more translucent pellicle (Fig. 1). In contrast, the *ex situ* method, in which preformed BC was immersed in an HC solution for 24 h, resulted in composites that appeared visually similar to pure BC, showing no obvious differences in surface appearance or texture. The dry weight of pure BC was approximately 0.35 g per 10 × 15 cm sheet. *In situ*-prepared BC/HC composites exhibited slightly lower dry weights (*e.g.*, ∼0.3 g for BC/HCin-1), likely due to alterations in microfibrillar assembly during biosynthesis in the presence of HC, which may reduce the amount of cellulose produced. Conversely, *ex situ*-prepared composites displayed a modest increase in dry weight (*e.g.*, ∼0.4 g for BC/HCex-1), which can be attributed to additional mass contributed by collagen deposited onto the BC matrix.

After TTO loading, the resulting BC/HC/TTO membranes appeared slightly opaque and exhibited a mildly oily surface with a characteristic TTO scent, confirming successful incorporation of the essential oil into the composite matrix.

### Characteristics of BC/HC composites

#### FTIR analysis

**Fig. 2:**
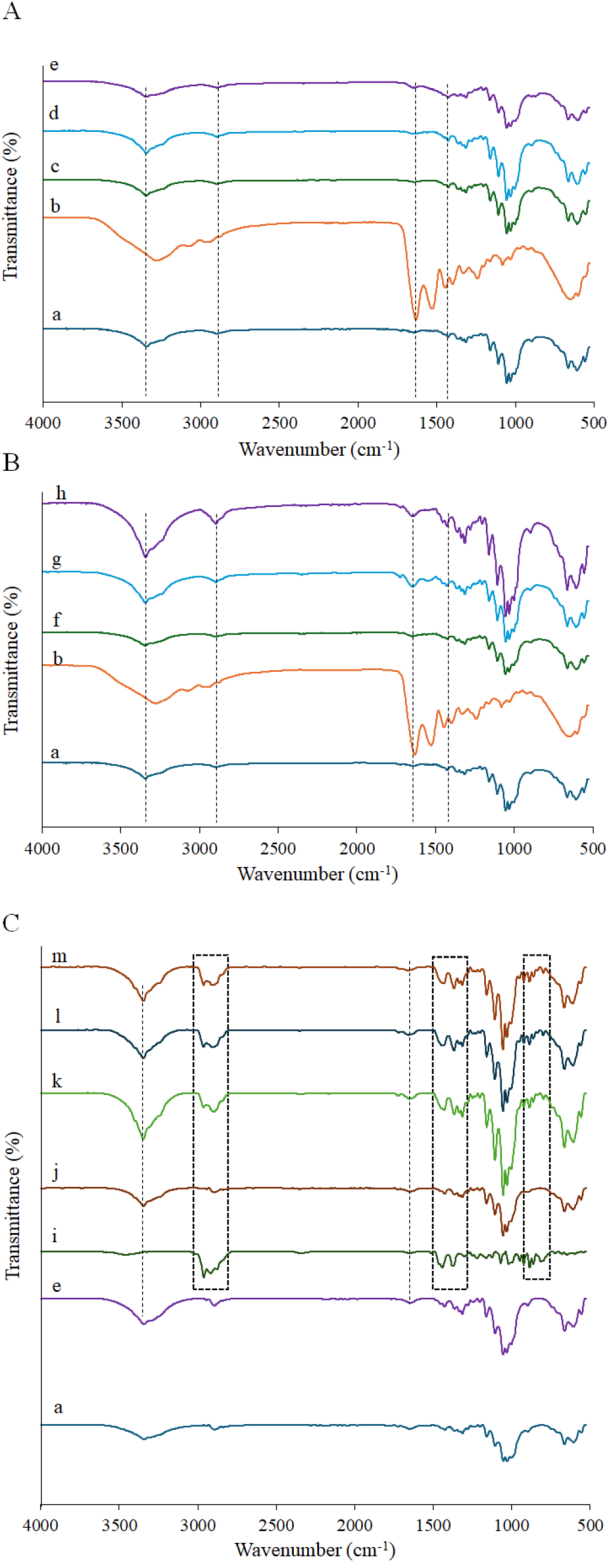
FTIR spectra of (A) BC/HC composites prepared by *in situ* method (a: BC, b: HC, c: BC/HCin-0.1, d: BC/HCin-0.5, e: BC/HCin-1), (B) BC/HC composites prepared by *ex situ* method (a: BC, b: HC, f: BC/HCex-0.1, g: BC/HCex-0.5, h: BC/HCex-1), and (C) BC/HC composites loaded with TTO (a: BC, e: BC/HCin-1), i: TTO, j: BC/HC/TTO-1, k: BC/HC/TTO-3, l: BC/HC/TTO-5, m: BC/HC/TTO-10.

FTIR spectroscopy was used to confirm the incorporation of HC into the BC network and to verify the successful loading of TTO into the composites. The FTIR spectra of pure BC and BC/HC composites produced *via in situ* and *ex situ* modification are presented in Fig. 2A and B. Pure BC exhibited the characteristic absorption bands, consistent with literature reports.^44,45^ HC showed typical collagen-related amide bands (amide i, ii, and iii), confirming its peptide-based structure.^46^ In BC/HC composites, the appearance and progressive intensification of amide I bands (∼1635–1637 cm^−1^) with increasing HC content, together with an enhanced broad O–H/N–H band around 3400 cm^−1^, indicate successful incorporation of HC and increased hydrogen bonding within the composite matrix. These features were more pronounced in *ex situ*-modified samples with higher HC content, reflecting differences in collagen distribution between the two modification approaches.

The FTIR spectrum of TTO (Fig. 2C) displayed characteristic bands associated with terpene-based functional groups.^47,48^ After TTO loading, both *in situ* and *ex situ* BC/HC composites exhibited new absorption bands corresponding to these TTO functional groups (*e.g.*, ∼2960, 1656, and 1112 cm^−1^), confirming the successful adsorption of TTO into the BC/HC matrix without alteration of the composite structure.

#### SEM analysis

**Fig. 3:**
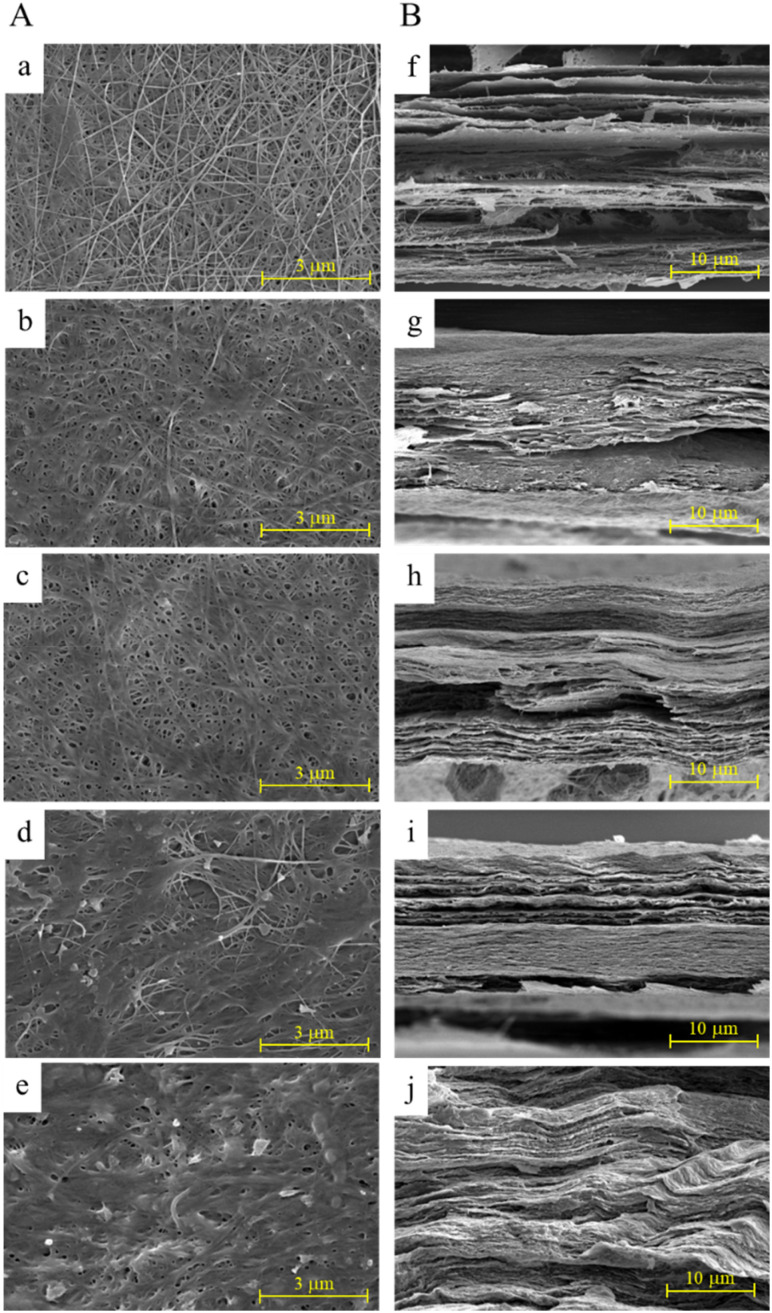
SEM images of (A) surface and (B) cross-section of pure BC (a–f); BC/HCin-0.1 (b–g); BC/HCin-1 (c–h); BC/HCex-0.1 (d–i); BC/HCex-1 (e–j). These images were taken at 200 00× and 5000× magnifications for surface and cross-section, respectively.

SEM imaging was used to examine the surface and cross-sectional morphologies of pure BC and BC/HC composites (Fig. 3). Pure BC exhibited a characteristic three-dimensional nanofibrous network with pore sizes of approximately 10–20 nm and a multilayered cross-sectional structure, consistent with previous reports.^30,49,50^*In situ* HC modification induced pronounced morphological changes, including enlarged surface pores and a lighter, more open internal network, which became more evident with increasing HC content. These observations suggest that HC incorporation during biosynthesis interfered with BC microfibril assembly, leading to altered fibrillation and reduced structural compactness.^51,52^ In contrast, *ex situ*-modified BC/HC composites showed smoother surfaces and reduced porosity, attributed to collagen deposition forming a uniform, compact coating over the BC network,^53^ as confirmed by cross-sectional images.

#### DSC analysis

**Fig. 4:**
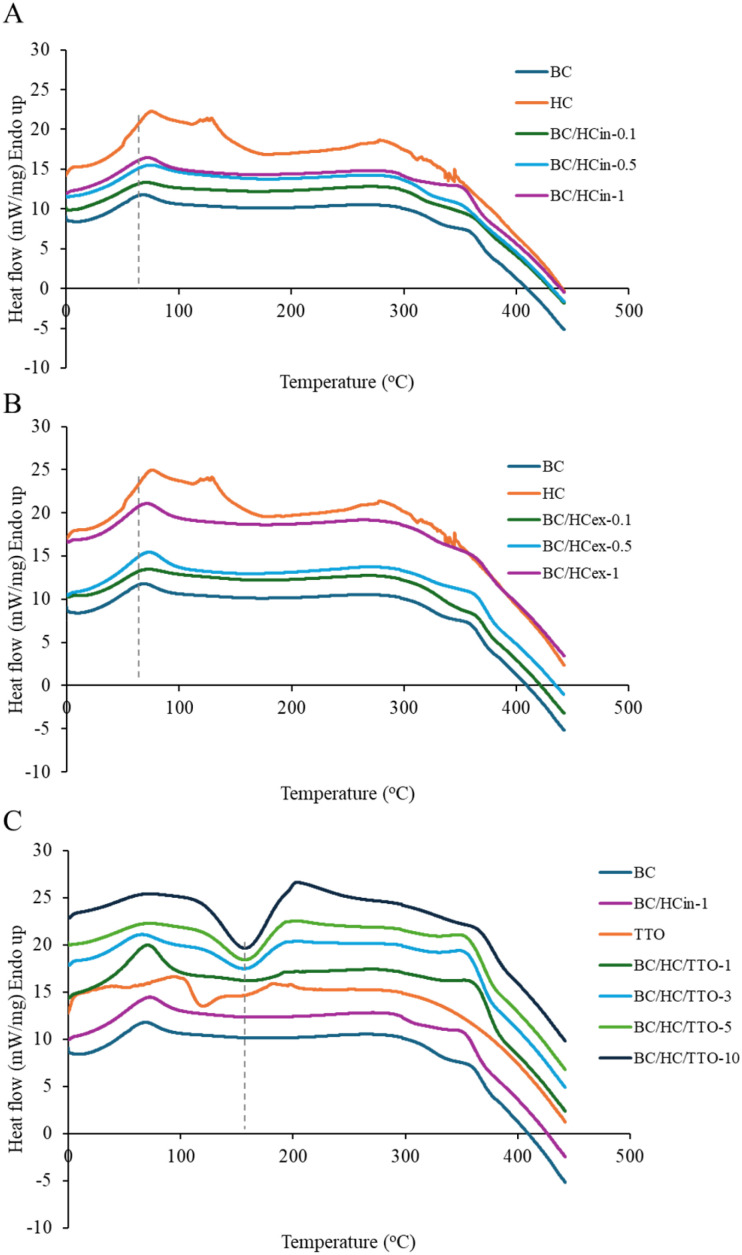
DSC thermograms of (A) BC/HC composites prepared by *in situ* method, (B) BC/HC composites prepared by *ex situ* method, and (C) BC/HC composites loaded with TTO.

The DSC thermograms of BC, HC, TTO, and their composites are presented in Fig. 4. Pure BC exhibited an initial endothermic event attributed to moisture loss, followed by thermal stability up to approximately 290 °C and a degradation-related transition above 300 °C, consistent with the high crystallinity and thermal resistance of bacterial cellulose.^44^ HC showed a broad endothermic transition between 70 and 150 °C associated with bound water removal, with a degradation peak near 260 °C corresponding to peptide-chain denaturation.^54,55^ Incorporation of HC into BC *via* both *in situ* and *ex situ* approaches resulted in a gradual shift of the composite endothermic transitions toward higher temperatures as HC content increased, indicating effective integration of collagen within the BC matrix. The slightly greater shift observed for *ex situ* composites is consistent with their higher surface-associated HC content, as supported by SEM observations.

The DSC profile of TTO displayed a broad endothermic peak below 120 °C due to evaporation of volatile components, followed by an exothermic peak around 140 °C attributed to oxidative reactions.^56^ In TTO-loaded BC/HC composites, the evaporation-related endothermic peak decreased in intensity, while the exothermic peak shifted to higher temperatures (∼160 °C) and broadened. These changes suggest interactions between TTO and the BC/HC matrix that partially suppress volatilization and delay oxidative degradation. Overall, the DSC results confirm successful composite formation and indicate that incorporation into the BC/HC network enhances the thermal stability of TTO.

#### Mechanical properties

**Fig. 5:**
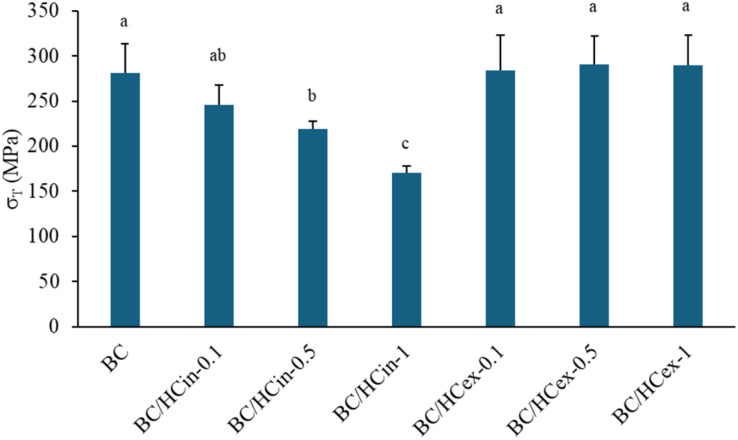
Tensile strength of BC and BC/HC composites prepared by *in situ* and *ex situ* methods using different amounts of HC. Data are presented as mean ± standard deviation from three independent experiments (*n* = 3). Difference in mean was analyzed using one-way ANOVA at significant level of 0.05; the same letter indicates that the mean values are not statistically different.

The tensile strengths of pure BC and BC/HC composites prepared *via in situ* and *ex situ* modification are shown in Fig. 5. Pure BC exhibited a high tensile strength of approximately 280 MPa, attributed to its highly interconnected nanofibrillar network and high crystallinity. *In situ* HC modification resulted in a progressive decrease in tensile strength with increasing HC content (*e.g.*, ∼170 MPa for BC/HCin-1), likely due to disruption of the crystalline cellulose structure and interference with microfibril assembly during biosynthesis, consistent with previous reports.^57^ In contrast, *ex situ* HC modification slightly increased tensile strength (approximately 284–290 MPa) with increasing collagen concentration, which can be attributed to collagen deposition reinforcing the BC network, in agreement with earlier studies.^58^

#### XRD analysis

**Fig. 6:**
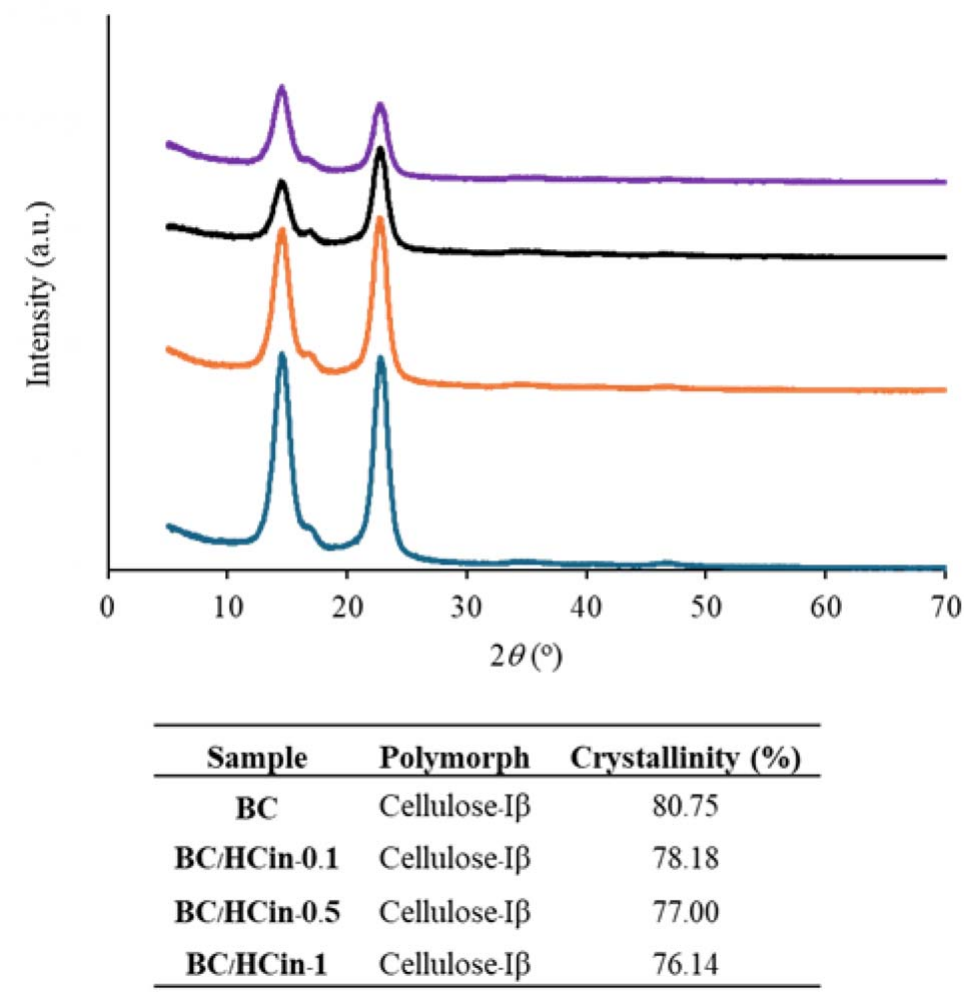
XRD patterns and crystallinity index of BC and BC/HC composites prepared by *in situ* method using different amounts of HC (a: BC, b: BC/HCin-0.1, c: BC/HCin-0.5, d: BC/HCin-1).

The XRD pattern of pure BC (Fig. 6) exhibited characteristic diffraction peaks at 2*θ* ≈ 14.5°, 16.2°, and 22.9°, corresponding to the (1–10), (110), and (200) planes, confirming its highly crystalline structure. After HC modification, the primary cellulose diffraction peaks were retained; however, peak broadening and reduced intensity were observed, becoming more pronounced with increasing HC content. Quantitative analysis showed a progressive decrease in crystallinity index with increasing HC incorporation, attributed to the presence of amorphous collagen and disruption of cellulose microfibril organization during *in situ* biosynthesis. These results are consistent with previous reports^57,59^ and indicate partial amorphization of BC/HC composites, which may be beneficial for applications requiring enhanced flexibility and improved loading of active agents.

#### Water holding capacity (WHC)

**Fig. 7:**
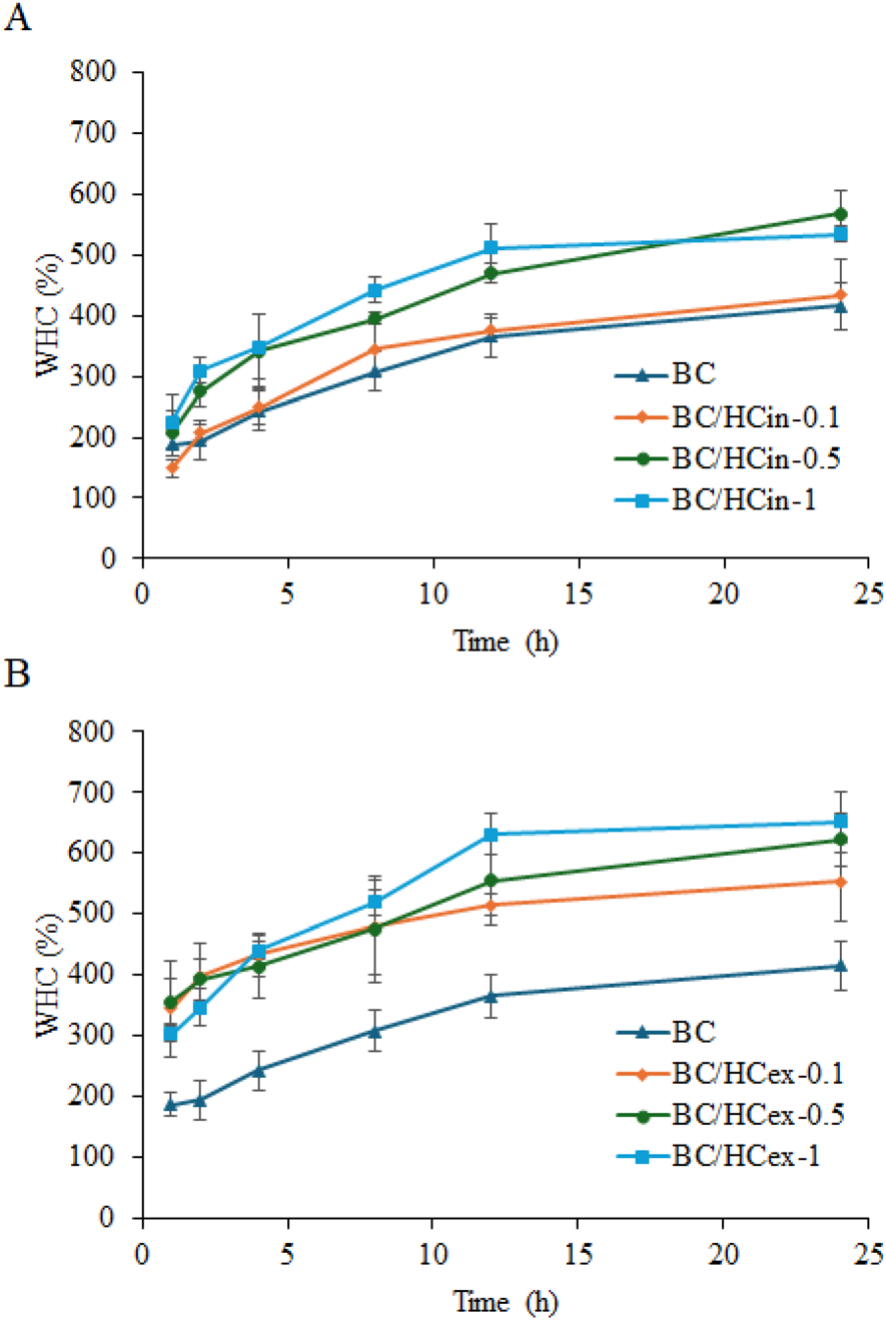
Water holding capacity (WHC) profiles of BC and BC/HC composites prepared by (A) *in situ* and (B) *ex situ* methods using different amounts of HC. Data are presented as mean ± standard deviation from three independent experiments (*n* = 3).

The WHC of pure BC and BC/HC composites after 24 h of water immersion is shown in Fig. 7. Pure BC exhibited the lowest WHC (∼250%), whereas both *in situ* and *ex situ* BC/HC composites showed significantly enhanced water absorption, increasing with collagen content. This improvement is attributed to the hydrophilic nature of HC and its interaction with the BC network. Notably, in situ-modified BC/HC composites exhibited higher WHC than *ex situ* counterparts, indicating more effective and homogeneous incorporation of HC within the BC matrix during biosynthesis. Similar enhancement of swelling behaviour has been reported for BC-based composites incorporating hydrophilic modifiers.^60^

#### Water retention value (WRV)

**Fig. 8:**
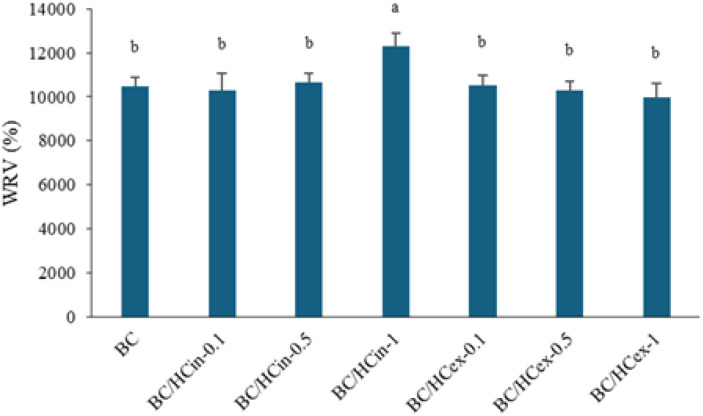
Water retention value (WRV) of BC and BC/HC composites prepared by *in situ* and *ex situ* methods using different amounts of HC. Data are presented as mean ± standard deviation from three independent experiments (*n* = 3). Difference in mean was analyzed using one-way ANOVA at significant level of 0.05; the same letter indicates that the mean values are not statistically different.

Pure BC exhibited excellent water retention, with a WRV of approximately 10 462% (Fig. 8), attributable to its highly porous three-dimensional network. *In situ* HC modification led to a slight increase in WRV with increasing HC content, with BC/HCin-1 showing the highest value, likely due to enhanced porosity and increased amorphous character,^61^ consistent with SEM and XRD results. In contrast, *ex situ* HC modification resulted in a decreasing trend in WRV with increasing HC content, possibly due to surface collagen deposition limiting water uptake; however, the WRV values of ex situ-modified samples were not significantly different from that of pure BC.

### TTO loading content

**Fig. 9:**
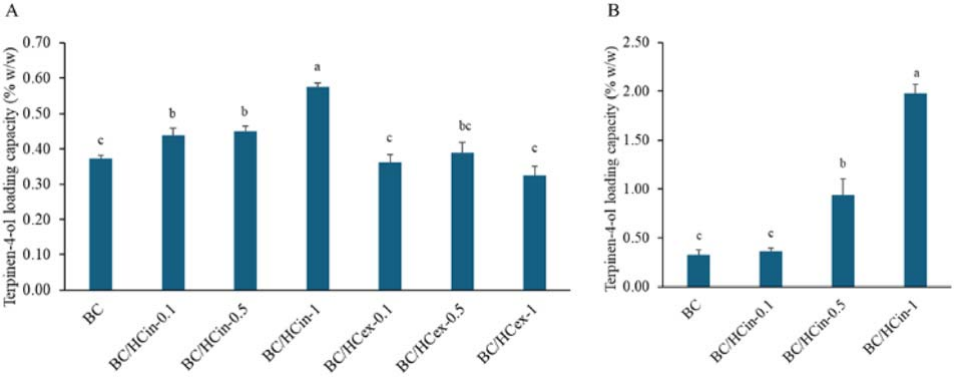
Loading capacity of TTO, expressed as terpinen-4-ol content and calculated based on the dry mass of the composite (% w/w), in BC and BC/HC composite prepared by (A) *in situ* and *ex situ* methods in dry form and (B) *in situ* method in wet form using different amounts of HC. Data are presented as mean ± standard deviation from three independent experiments (*n* = 3). Difference in mean was analyzed using one-way ANOVA at significant level of 0.05; the same letter indicates that the mean values are not statistically different.

Fig. 9 presents the loading capacity of TTO in BC/HC composites under both dried and wet loading conditions, expressed as terpinene-4-ol content relative to the dry mass of the composite (% w/w), enabling standardized comparison across all samples. In the dried state, the BC/HCin composite exhibited the highest TTO loading capacity (0.56% w/w), compared to BC/HCex composite (0.36% w/w) and unmodified BC (0.34% w/w) (Fig. 9A). These results indicate that *in situ* modification significantly enhances the affinity of the composite for TTO compared with *ex situ* modified and unmodified BC. As all samples were standardized in terms of size and dry mass prior to loading, the observed improvement can be attributed to the formation of smaller fibrils and the associated increase in specific surface area and porosity, along with a reduction in crystallinity; these structural changes promote greater adsorption and retention of TTO within the cellulose network.

For wet state loading, only the *in situ* composites were evaluated, as they had already shown superior loading performance in the dried state. In the wet state, *in situ* modified BC displayed a markedly higher TTO loading efficiency, incorporating more than twice the amount of TTO compared to the dried form (Fig. 9B). This enhancement is likely due to the preservation of the hydrated, porous, and flexible structure of the cellulose matrix, which remains more accessible to hydrophobic molecules such as TTO. In contrast, the drying process collapses pore structures and increases matrix rigidity, thereby restricting TTO penetration and limiting its retention within the BC network.

These findings suggest that maintaining the composite in a partially hydrated state prior to loading markedly improves TTO incorporation efficiency. Based on this enhanced performance, the wet BC/HCin-1 composite was selected for subsequent TTO loading, and the resulting TTO-loaded samples were used in release studies, antibacterial activity assessments, time-kill kinetics, and stability evaluations.

### *In vitro* release study of TTO-loaded BC/HC composites

**Fig. 10:**
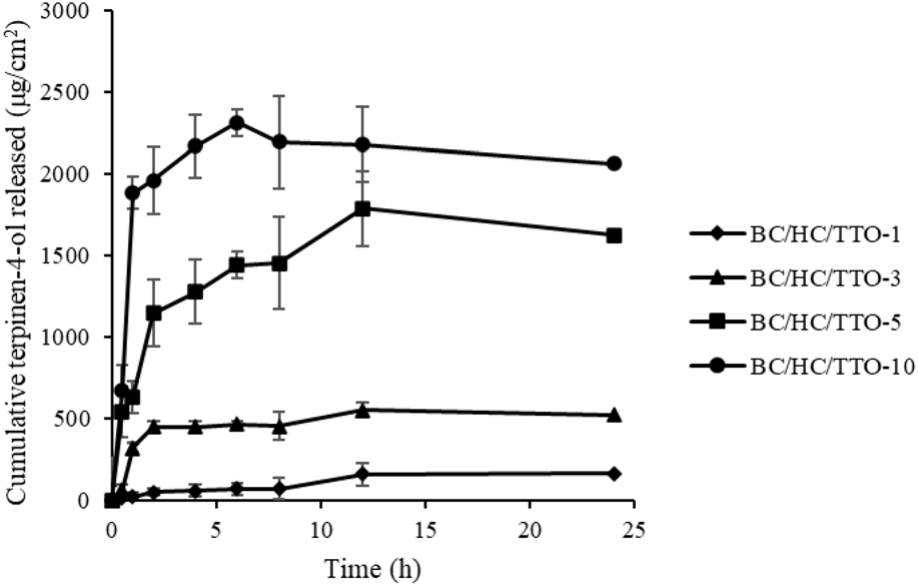
Cumulative release of terpinen-4-ol from BC/HC composite loaded with different concentrations of TTO. Data are presented as mean ± standard deviation from three independent experiments (*n* = 3).

Fig. 10 presents the *in vitro* release profile of terpinen-4-ol from TTO-loaded BC/HC composites. All samples exhibited a biphasic release pattern, consisting of an initial rapid release within the first 1–2 h, followed by a slower, sustained release from approximately 2–8 h until equilibrium was reached. The initial burst release is attributed to the diffusion of TTO located on or near the surface of the modified BC film, in agreement with previous reports.^62^

This biphasic release behaviour, rapid initial delivery followed by prolonged release, demonstrates the potential of TTO-loaded BC/HC composites for biomedical applications, such as wound healing and topical antibacterial therapy, where both immediate and sustained bioactive effects are desirable.

### Antibacterial activity analysis

**Fig. 11:**
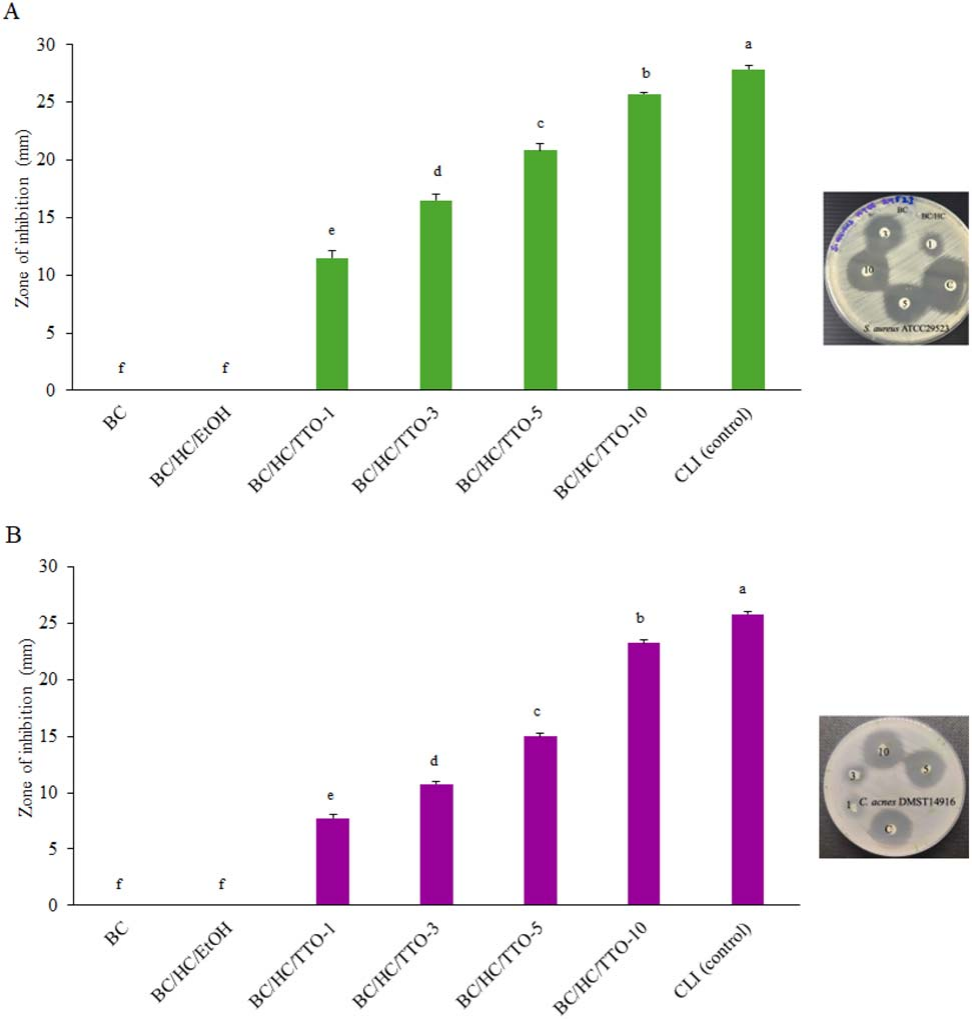
Zone of inhibition of BC, BC/HC/EtOH (vehicle control), and BC/HC composite membranes loaded with different concentrations of TTO against (A) *S. aureus* and (B) *C. acnes* compared with clindamycin (CLI). Data are presented as mean ± standard deviation from three independent experiments (*n* = 3). Difference in mean was analyzed using one-way ANOVA at significant level of 0.05; the same letter indicates that the mean values are not statistically different.

The antibacterial activity of BC, vehicle control (BC/HC/EtOH), and TTO-loaded BC/HC films was evaluated using the disc diffusion assay, and the results are presented in Fig. 11A and B. Unmodified BC and BC/HC/EtOH did not exhibit any measurable zones of inhibition against either bacterial strain, confirming the absence of inherent antibacterial activity and excluding any contribution from residual ethanol. This observation is consistent with previous findings by Yakaew *et al.* (2022),^37^ confirming the inherent biocompatibility of BC-based materials without incorporated antibacterial agents.

In contrast, TTO-loaded BC/HC composites displayed clear zones of inhibition against both *S. aureus* and *C. acnes*, with ZOI diameters increasing progressively with higher TTO loadings ranging from 1% to 10%. As all samples were standardized in terms of disc size, the observed enhancement in antibacterial activity can be directly attributed to increased TTO content and its diffusion from the composite matrix into the agar medium. The BC/HC/TTO-10 composite exhibited comparable activity to the CLI positive control, validating the effectiveness of the formulation and the reliability of the assay in accordance with CLSI recommendation.

These results indicate that the antibacterial efficacy of BC/HC/TTO films is directly proportional to the amount of TTO incorporated into the BC matrix. Similar trends have been reported for BC membranes loaded with bacitracin or amoxicillin, which showed enhanced antibacterial activity against *E. coli* (ATCC 25922) and *S. aureus* (ATCC 25923).^63^ The absence of activity in unmodified BC and BC/HC underscores their suitability as biocompatible carriers capable of controlled release, whereas pure TTO tends to evaporate rapidly.

The antibacterial effect of TTO within the BC/HC matrix is primarily attributed to terpinen-4-ol, along with other monoterpenes, which disrupt bacterial cell membranes, increase permeability, and induce leakage of intracellular components.^64^ These findings suggest that TTO-loaded BC/HC composites could serve as a safer and more sustainable alternative to conventional antibiotics, offering potential applications in biomedical fields such as acne treatment and the management of wound infections, while contributing to strategies against antibiotic resistance.

### Time-kill kinetics

**Fig. 12:**
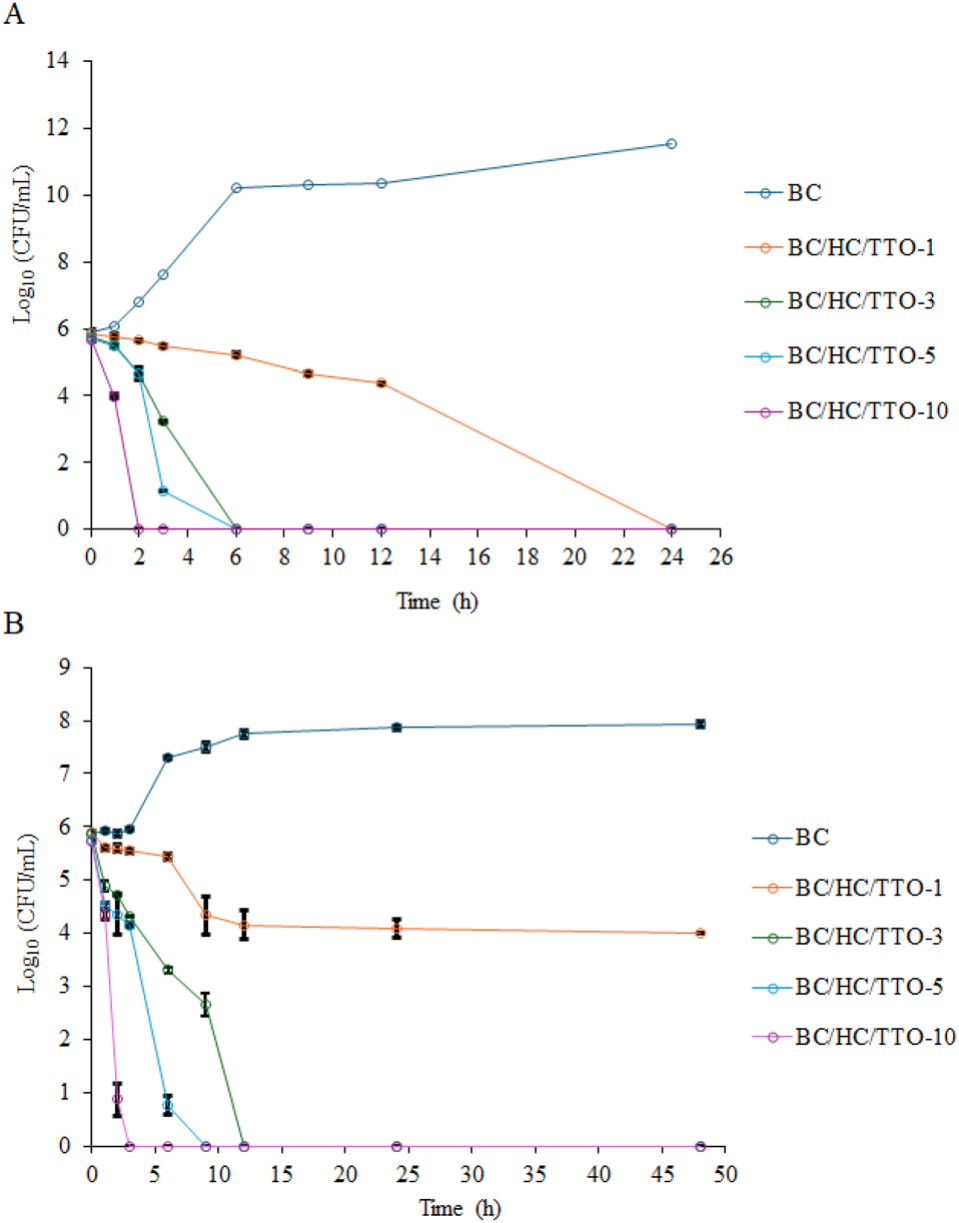
Time-kill kinetics of pure BC and BC/HC composite loaded with different concentrations of TTO against (A) *S. aureus* and (B) *C. acnes.* Viable bacterial counts are expressed as log_10_ CFU mL^−1^. Data are presented as mean ± standard deviation from three independent experiments (*n* = 3). Pure BC served as the untreated control.

The time-kill kinetics assay was performed to evaluate the antibacterial activity of BC and BC/HC/TTO composites against *S. aureus* and *C. acnes* at various time intervals, and the results are presented in Fig. 12. At all sampling time points, viable bacterial counts were quantified as CFU mL^−1^ using the same dilution scheme and a fixed plated volume of 100 µL. Time points at which no colonies were detected correspond to bacterial counts below the assay detection limit. For *S. aureus* (Fig. 12A), pure BC exhibited no antibacterial effect, with bacterial growth increasing exponentially from 2 to 12 h, reaching approximately 4 log_10_ CFU mL^−1^, and entering a stationary phase with counts exceeding 12 log_10_ CFU mL^−1^ by 24 h. This observation is consistent with previous studies reporting that unmodified BC lacks inherent antibacterial activity and that antimicrobial effects arise only after incorporation of essential oils.^65^ In contrast, BC loaded with 1% TTO solution showed a slight increase in bacterial growth during the first 6 h, followed by a gradual decline, resulting in no detectable colonies at 24 h. At 3% TTO loading solution, a slower reduction in viable cells was observed up to 12 h before achieving complete elimination at 24 h. A more pronounced bactericidal effect was observed for 5% TTO loading solution, with a significant reduction within 6 h and complete bacterial elimination thereafter. The BC/HC/TTO-10 composite exhibited the fastest and most sustained antibacterial effect, achieving no detectable colonies within 2 h. These findings are in agreement with the study by Manzanelli *et al.* (2023), who reported that pure TTO at 0.2% (v/v) significantly reduced *S. aureus* viability and maintained suppression over 24 h.^66^ These results indicate a strong, concentration-dependent bactericidal activity of BC/HC/TTO composites against *S. aureus*, with reductions exceeding 3 log_10_ CFU mL^−1^. This rapid bactericidal action aligns with the *in vitro* release data, which showed a pronounced initial burst release within the first 2 h. The concordance between release characteristic and bacterial reduction suggests that the composite can deliver an effective early antibacterial dose while maintaining sustained activity when applied to infected skin.

For *C. acnes* (Fig. 12B), pure BC showed exponential bacterial growth between 3 and 6 h, followed by a slight decline; thereafter, counts steadily increased from 12 to 48 h, reaching approximately four times the initial population, confirming that BC alone does not inhibit C. acnes. The BC/HC/TTO-1 composite (using 1% TTO loading solution) suppressed bacterial growth during the first 6 h, followed by a gradual decline, resulting in an approximate 2 log_10_ CFU mL^−1^ reduction by 48 h, indicative of a bacteriostatic effect. Composites loaded with 3% and 5% TTO loading solution exhibited progressive reductions in bacterial counts, achieving complete eradication by 24 h, demonstrating a bactericidal effect. Notably, the BC/HC/TTO-10 composite showed the most rapid and sustained antibacterial action, completely eliminating *C. acnes* within 2 h with no regrowth observed over 48 h. This rapid killing at higher TTO loadings is consistent with the findings of Esmael *et al.* (2020), who reported complete elimination of *C. acnes* within 4 h in time-kill kinetic studies using TTO.^67^

These findings confirm that TTO incorporation into BC enhances its antibacterial efficacy in a concentration-dependent manner, with higher TTO loadings providing faster and more effective bactericidal activity against both Gram-positive pathogens. However, it should be noted that these experiments were conducted using planktonic, actively growing bacteria in sebum-free media. Consequently, the antibacterial activity observed here may not fully reflect acne-relevant conditions, where reduced bacterial growth rates, biofilm formation, and the presence of sebum-derived lipids may diminish monoterpene availability and antimicrobial efficacy.

### Stability of TTO-loaded BC/HC composites

**Fig. 13:**
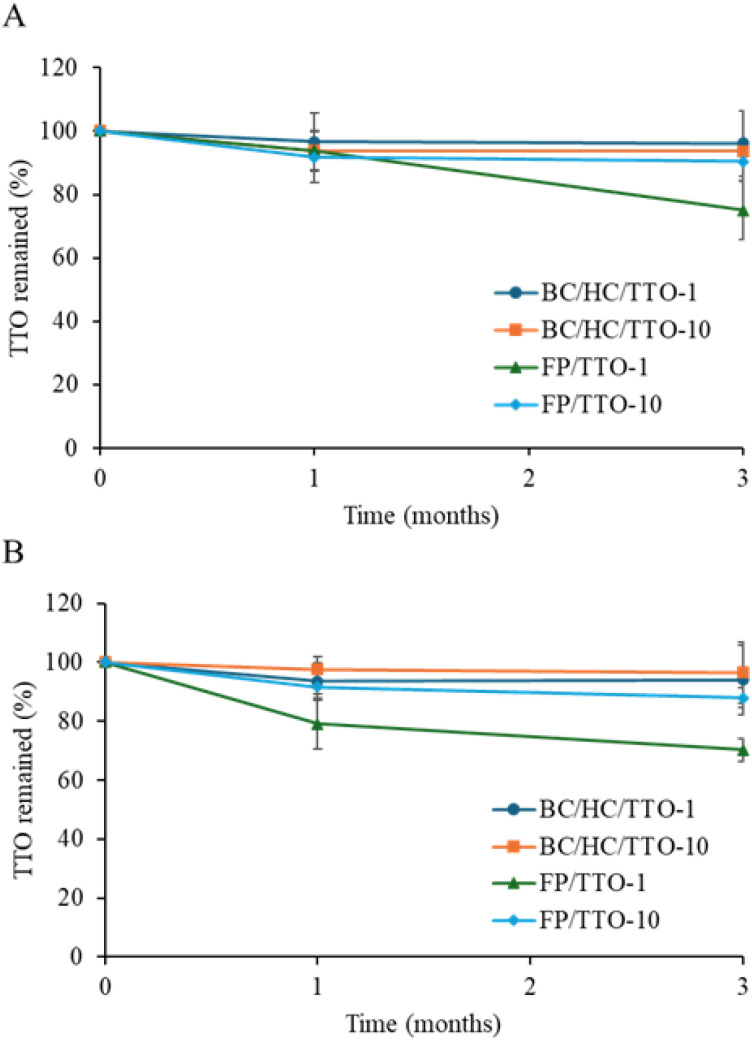
The percentage of TTO remained in BC/HC composite membranes loaded with low (1%) and high (10%) TTO concentrations after storage at (A) normal (30 ± 5 °C/75 %RH) and (B) accelerated (40 ± 5 °C/75 %RH) conditions over a period of 0 to 3 months compared to filter paper (FP) loaded with the same concentration of TTO. Data are presented as mean ± standard deviation from three independent experiments (*n* = 3).

Stability assessment is essential for ensuring the long-term efficacy of active compounds, particularly those susceptible to degradation. Although essential oils such as TTO possess significant therapeutic potential, they are susceptible to volatilization and oxidative degradation when exposed to environmental factors. To evaluate the stability of TTO incorporated within the BC/HC matrix, composites loaded with low (1% TTO loading solution) and high (10% TTO loading solution) concentrations were stored for three months at 30 °C and 40 °C. Stability was assessed by quantifying retained TTO content and monitoring antibacterial activity. As shown in Fig. 13, BC/HC composites loaded with 1% TTO exhibited high stability at 30 °C, retaining 96.5% of the initial TTO after one month and 96.0% after three months. At 40 °C, retention remained similarly high, at 93.4% and 94.0% after one and three months, respectively. For BC/HC composites loaded with 10% TTO, retention at 30 °C was 93.0% after one month and 93.6% after three months, while samples stored at 40 °C maintained 97.0% and 96.0% retention over the same intervals. All composites preserved more than 90% of their initial TTO content after three months, confirming that the BC/HC matrix effectively stabilizes TTO by minimizing volatilization and thermal degradation.

In contrast, TTO loaded onto filter paper exhibited substantially lower stability, highlighting the protective role of the BC/HC matrix. Overall, only minimal TTO loss was observed in BC/HC composites, demonstrating that the bacterial cellulose–collagen network effectively stabilizes the oil, preventing volatilization and degradation even under accelerated storage conditions. These results underscore the potential of BC/HC as a reliable delivery matrix for maintaining the bioactivity of volatile therapeutic oils over extended periods.

The retained antibacterial activity of 1% and 10% TTO-loaded BC/HC composites was also evaluated over a three-month storage period at 30 ± 2 °C/75% RH and 40 ± 2 °C/75% RH, using Day 0 as the baseline value (100% activity) (Table 1). A gradual decline in antibacterial efficacy was observed. After one month, both TTO-loaded BC/HC composites retained high antibacterial activity, exhibiting less than 20% loss against both *S. aureus* and *C. acnes* under most storage conditions. An exception was observed for BC/HC/TTO-1 stored at 40 ± 2 °C/75% RH, which exhibited complete loss of activity against *C. acnes*. By three months, BC/HC/TTO-10 maintained antibacterial activity with losses remaining below approximately 30% under both storage conditions, indicating good preservation of antibacterial function. In contrast, BC/HC/TTO-1 retained partial activity against *S. aureus* when stored at 30 ± 2 °C/75% RH but showed complete loss of activity against *C. acnes* under both storage conditions. By comparison, TTO loaded onto filter paper (FP) exhibited markedly lower stability, with complete loss of activity for 1% TTO-FP and more than 70% activity loss for 10% TTO-FP against both bacterial strains under both conditions.

**Table 1:** Stability studies of retained activity of BC/HC composite membranes loaded with low (1%) and high (10%) TTO concentrations against selected pathogens after storage at normal (30 ± 5 °C/75 %RH) and accelerated (40 ± 5 °C/75 %RH) conditions over a period of 0 to 3 months compared to filter paper (FP) loaded with the same concentration of TTO. Data are presented as mean ± standard deviation from three independent experiments (*n* = 3)

Storage conditions	Sample	Storage time (months)	Retained activity (%)	
			*S. aureus*	*C*. *acnes*
30 ± 5 °C/75% RH	BC/HC/TTO-1	0	100 ± 0.00	100 ± 0.00
		1	71.9 ± 2.74	89.09 ± 1.97
		3	54.43 ± 1.78	0.00 ± 0.00
	FP/TTO-1	1	0.00 ± 0.00	0.00 ± 0.00
		3	0.00 ± 0.00	0.00 ± 0.00
	BC/HC/TTO-10	0	100 ± 0.00	100 ± 0.00
		1	93.56 ± 2.34	86.70 ± 1.42
		3	66.60 ± 3.78	72.48 ± 1.56
	FP/TTO-10	1	31.12 ± 2.17	25.93 ± 1.50
		3	24.14 ± 2.61	0.00 ± 0.00
40 ± 5 °C/75% RH	BC/HC/TTO-1	0	100 ± 0.00	100 ± 0.00
		1	61.25 ± 0.93	0.00 ± 0.00
		3	0.00 ± 0.00	0.00 ± 0.00
	FP/TTO-1	1	0.00 ± 0.00	0.00 ± 0.00
		3	0.00 ± 0.00	0.00 ± 0.00
	BC/HC/TTO-10	0	100 ± 0.00	100 ± 0.00
		1	91.57 ± 2.91	81.52 ± 7.65
		3	64.39 ± 4.86	69.91 ± 2.94
	FP/TTO-10	1	20.59 ± 0.76	24.63 ± 0.67
		3	0.00 ± 0.00	0.00 ± 0.00

These results demonstrate that incorporation of TTO into the BC/HC composite significantly enhances its chemical and functional stability. Although more than 90% of TTO was retained during storage, a partial reduction in antibacterial activity was observed, demonstrating that chemical retention alone does not fully predict antibacterial performance. The antibacterial efficacy of TTO depends on the combined activity and bioavailability of multiple constituents rather than solely on terpinen-4-ol.^67,68^ The porous nanofiber architecture of BC/HC likely contributes to this effect by effectively entrapping TTO within the matrix and limiting volatilization and oxidative degradation over time. These characteristics establish BC/HC as a promising delivery vehicle for maintaining the stability and bioactivity of volatile therapeutic oils in topical applications.

## Conclusions

In this study, a novel tea tree oil (TTO) delivery system based on bacterial cellulose modified with hydrolyzed collagen (BC/HC) was successfully developed using both *in situ* and *ex situ* approaches and comprehensively evaluated for its suitability as a topical antimicrobial platform against acne-associated bacteria. In line with the study objectives, the two collagen modification strategies were systematically investigated, revealing that *in situ* modification more effectively altered the BC microstructure by reducing crystallinity, increasing porosity, and enhancing water-holding capacity. These structural and physicochemical changes led to a substantially higher TTO loading capacity, particularly when the composite was maintained in a swollen state prior to loading.

The BC/HC composites effectively incorporated and retained TTO, as confirmed by FTIR, DSC, and GC-based analyses, while also providing partial thermal and oxidative protection to the volatile oil. *In vitro* release studies demonstrated a desirable biphasic release profile, consisting of an initial burst followed by sustained release, which is well suited for topical antibacterial applications requiring both rapid onset and prolonged activity. TTO-loaded BC/HC composites exhibited strong, loading-dependent antibacterial activity against *S. aureus* and *C. acnes*, with no detectable colonies at higher TTO loadings confirmed by disc diffusion and time-kill kinetic assays. Stability studies further demonstrated that incorporation of TTO into the BC/HC matrix significantly enhanced its chemical and functional stability, with more than 90% TTO retained after three months under both normal and accelerated storage conditions, alongside preservation of antibacterial efficacy at one month for both tested strains. This performance was markedly superior to that of TTO applied to conventional substrates, highlighting the protective role of the BC/HC nanofibrous network against volatilization and degradation.

Overall, these findings indicate that HC-modified BC, particularly when prepared *via in situ* modification, represents a promising, natural, and sustainable biopolymer-based delivery platform for TTO. By combining high loading efficiency, controlled release, robust antibacterial activity, and improved stability, the BC/HC/TTO composite shows strong potential as a topical antimicrobial platform active against acne-associated bacteria. Nevertheless, the present study is limited to *in vitro* antibacterial evaluations, which do not fully replicate the complex pilosebaceous environment of acne, including sebum-rich conditions and biofilm formation that may influence diffusion and antimicrobial efficacy. Future studies should therefore focus on evaluating BC/HC/TTO composites under acne-relevant conditions, including anti-biofilm and anti-inflammatory assays, as well as *in vivo* skin compatibility, irritation potential, and clinical efficacy to further validate the translational potential of this system for dermatological applications.

## Author contributions

T. S.: methodology, investigation, formal analysis, visualization, data curation, writing – original draft, writing – review & editing. J. J.: methodology, formal analysis, visualization, writing – review & editing. S. K.: methodology, formal analysis, visualization, writing – review & editing. N. N.: methodology, formal analysis, visualization, writing – review & editing. C. J.: conceptualization, methodology, visualization, supervision, writing – review & editing. All authors approved the final version of the manuscript before submission.

## Conflicts of interest

There are no conflicts to declare.

## Supplementary Material

RA-016-D5RA09816E-s001

## Data Availability

The supporting data has been provided as part of the supplementary information (SI). Supplementary information: Fig. S1 and S2 (SEM surface morphology and comparison of zones of inhibition) and Tables S1–S3 (detailed results of zone of inhibition measurements and time–kill kinetics assays). See DOI: https://doi.org/10.1039/d5ra09816e.
