# Supplementary material for: Hydrolyzed collagen-modified bacterial cellulose loaded with tea tree oil for antibacterial activity against acne-associated bacteria
Source: RSC Adv. 2026 Feb 17;16(10):9307–20. doi: 10.1039/d5ra09816e (PMC12910439; doi:10.1039/d5ra09816e)
Supplement: RA-016-D5RA09816E-s001 [file RA-016-D5RA09816E-s001.pdf]

Supplementary Information (SI) for RSC Advances.
This journal is © The Royal Society of Chemistry 2026

Supplementary Information

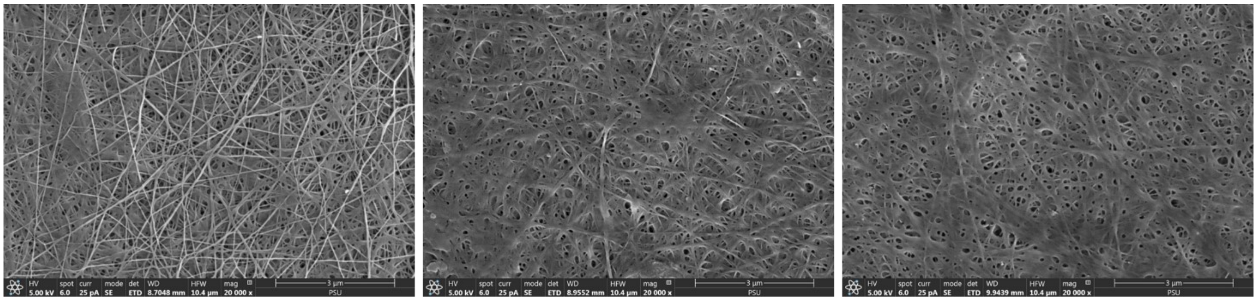

Pure BC BC/HCin-0.1 BC/HCin-1

Fig. S1 Surface morphology comparison of pure BC, BC/HCin-0.1, and BC/HCin-1 at the same magnification (20,000×) reveals a progressive reduction in fibril size with increasing HC content. Conversely, membrane pores become more clearly defined as the HC content increases.

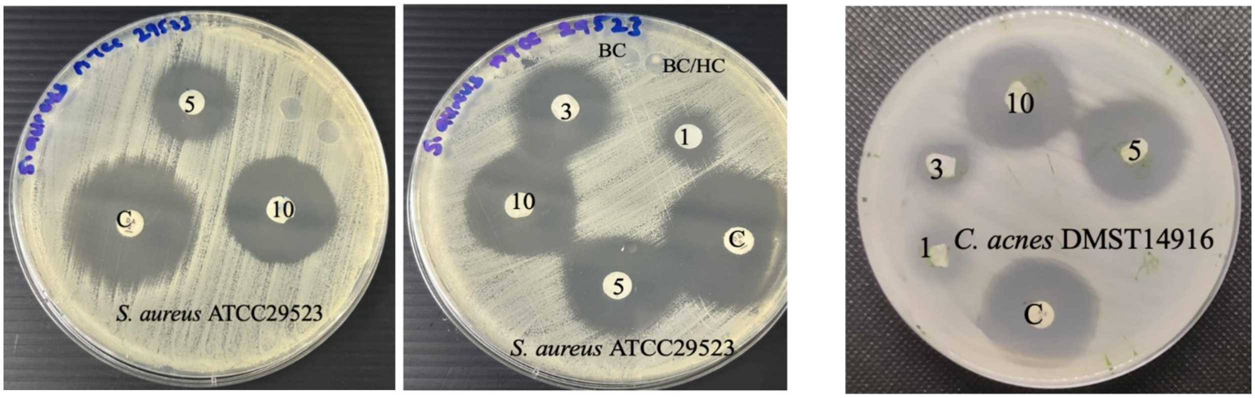

S. aureus *C. acnes*

Fig. S2 Disc diffusion assay results show clear zones of inhibition (ZOI) for BC, BC/HC, BC/HC/TTO-1, BC/HC/TTO-3, BC/HC/TTO-5, and BC/HC/TTO-10 against *Staphylococcus aureus* and *Cutibacterium acnes*.

Table S1 Zone of inhibition (ZOI) for BC, BC/HC, BC/HC/TTO-1, BC/HC/TTO-3, BC/HC/TTO-5, BC/HC/TTO-10, and clindamycin (CLN, positive control) against *Staphylococcus aureus* and *Cutibacterium acnes*

Sample	ZOI (mm)	
	S. aureus	*C. acnes*
BC	0.00 ± 0.00	0.00 ± 0.00
BC/HC	0.00 ± 0.00	0.00 ± 0.00
BC/HC/TTO-1	11.47 ± 0.65	7.75 ± 0.32
BC/HC/TTO-3	16.48 ± 0.60	10.78 ± 0.21
BC/HC/TTO-5	20.85 ± 0.57	14.96 ± 0.31
BC/HC/TTO-10	25.70 ± 0.19	23.20 ± 0.28
CLN (control)	27.85 ± 0.39	25.79 ± 0.28

Table S2 Time-kill kinetics of BC/HC composites loaded with TTO at different amounts against *Staphylococcus aureus*. TTO incorporation into BC/HC composites enhances its antibacterial efficacy in a dose-dependent manner, with higher TTO loadings providing faster and more effective bactericidal activity.

Time (h)	Log_{10} CFU/mL of *S. aureus*				
	BC	BC/HC/TTO-1	BC/HC/TTO-3	BC/HC/TTO-5	BC/HC/TTO-10
0	5.880±0.06	5.834±0.14	5.769±0.08	5.728±0.09	5.655±0.04
1	6.081±0.06	5.757±0.07	5.525±0.06	5.486±0.04	3.996±0.07
2	6.819±0.03	5.646±0.02	4.643±0.01	4.674±0.19	0.000±0.00*
3	7.612±0.07	5.490±0.04	3.229±0.04	0.000±0.00*	0.000±0.00*
6	10.215±0.01	5.226±0.08	0.000±0.00*	0.000±0.00*	0.000±0.00*
9	10.311±0.02	4.674±0.04	0.000±0.00*	0.000±0.00*	0.000±0.00*
12	10.337±0.03	4.054±0.02	0.000±0.00*	0.000±0.00*	0.000±0.00*
24	12.086±0.04	0.000±0.00	0.000±0.00*	0.000±0.00*	0.000±0.00*
48	5.880±0.06	0.000±0.00	0.000±0.00*	0.000±0.00*	0.000±0.00*

* No bacterial growth detected (0 CFU).

Table S3 Time-kill kinetics of BC/HC composites loaded with TTO at different amounts against *Cutibacterium acnes*. TTO incorporation into BC/HC composites enhances its antibacterial efficacy in a dose-dependent manner, with higher TTO loadings providing faster and more effective bactericidal activity.

Time (h)	Log_{10} CFU/mL of *C. acnes*				
	BC	BC/HC/TTO-1	BC/HC/TTO-3	BC/HC/TTO-5	BC/HC/TTO-10
0	5.869±0.05	5.880±0.03	5.876±0.04	5.756±0.02	5.721±0.03
1	5.923±0.04	5.604±0.04	4.896±0.10	4.516±0.06	4.356±0.09
2	5.858±0.06	5.591±0.07	4.724±0.03	4.360±0.39	2.301±0.30
3	5.956±0.04	5.547±0.04	4.318±0.01	4.155±0.04	0.000±0.00*
6	7.300±0.04	5.435±0.05	3.305±0.06	2.100±0.17	0.000±0.00*
9	7.513±0.09	4.333±0.35	2.661±0.22	0.000±0.00*	0.000±0.00*
12	7.747±0.07	4.159±0.28	0.000±0.00*	0.000±0.00*	0.000±0.00*
24	7.870±0.04	4.100±0.17	0.000±0.00*	0.000±0.00*	0.000±0.00*
48	7.940±0.06	4.000±0.01	0.000±0.00*	0.000±0.00*	0.000±0.00*

* No bacterial growth detected (0 CFU).
